# From fever to action: diagnosis, treatment, and prevention of acute undifferentiated febrile illnesses

**DOI:** 10.1093/femspd/ftae006

**Published:** 2024-04-13

**Authors:** Muttiah Barathan

**Affiliations:** Department of Medical Microbiology, Faculty of Medicine, Universiti Malaya, 50603 Kuala Lumpur, Malaysia

**Keywords:** acute, febrile, respiratory, unidentified, and delayed

## Abstract

Acute Undifferentiated Febrile Illness (AUFI) presents a clinical challenge, often characterized by sudden fever, non-specific symptoms, and potential life-threatening implications. This review highlights the global prevalence, types, challenges, and implications of AUFI, especially in tropical and subtropical regions where infectious diseases thrive. It delves into the difficulties in diagnosis, prevalence rates, regional variations, and potential causes, ranging from bacterial and viral infections to zoonotic diseases. Furthermore, it explores treatment strategies, preventive measures, and the critical role of the One Health approach in addressing AUFI. The paper also addresses the emerging zoonotic risks and ongoing outbreaks, including COVID-19, Rickettsia spp., and other novel pathogens, emphasizing their impact on AUFI diagnosis and management. Challenges in resource-limited settings are analyzed, highlighting the need for bolstered healthcare infrastructure, enhanced diagnostics, and collaborative One Health strategies. Amidst the complexity of emerging zoonotic threats, this review underscores the urgency for a multifaceted approach to mitigate the growing burden of AUFI, ensuring early diagnosis, appropriate treatment, and effective prevention strategies.

## Teaser

Unveiling Acute Undifferentiated Febrile Illness: Global Prevalence, Challenges, and One Health Strategies for Emerging Zoonotic Threats in Resource-Limited Settings.

## Acute undifferentiated febrile illness (AUFI)

Undifferentiated febrile illness (UFI) is a common clinical presentation in healthcare settings worldwide. Patients with acute undifferentiated febrile illness (AUFI) often seek medical attention due to fever and other nonspecific symptoms (Wangdi et al. 2019). It is essential for healthcare professionals to accurately diagnose and manage AUFI to provide appropriate care. AUFI is a medical condition characterized by a sudden onset of fever (≥38°C or ≥ 100.4°F) that lasts for less than 2 weeks and cannot be attributed to a specific cause after a thorough clinical evaluation and appropriate laboratory testing (Shrestha et al. 2020). Acute febrile illness (AFI) is marked by the abrupt and rapid development of fever with other symptoms such as chills, headache, muscle aches, fatigue, and malaise. The fever may appear suddenly without warning and may not be preceded by any noticeable symptoms. The fever is associated with a range of non-specific symptoms, such as headache, body aches, chills, sweating, fatigue, cough, sore throat, gastrointestinal disturbances, and others. It may resolve on its own within a few days or may persist for up to two weeks (Shrestha et al. 2018). AFI is a common clinical presentation and can be caused by a wide range of infectious and non-infectious conditions. AFI can be a sign of serious illness and can lead to further complications if left untreated (Khirikoekkong et al. 2023). AFI is often the first indication that a person may have a more serious condition, early diagnosis is essential for isolating and treating the patient, which helps prevent the spread of the disease to others and can be crucial in the context of outbreaks (Elven et al. 2020). AFI can result from a wide range of underlying causes, including infections, inflammatory disorders, autoimmune diseases, and malignancies. Some of these conditions can be life-threatening if not properly managed. Many serious infections, such as sepsis, bacterial endocarditis, and certain viral infections, can present with a sudden high fever as a primary symptom. Without prompt intervention, these infections can lead to kidney, liver, and heart failure eventually lead to brain damage and death (Wainaina et al. 2022). For instance, untreated bacterial infections can result in abscess formation, which is a localized collection of pus that forms as a result of the body's immune response to infection. Abscesses can develop in various parts of the body, such as the skin, internal organs, or within tissues. They are typically characterized by symptoms such as localized pain, redness, swelling, warmth, and sometimes fever (Brook 2004).

### AUFI and types

AUFI can be broadly categorised into three sub-types, based on the signs and severity of the illness, the diagnosed acute febrile illness (Diagnosed AFI), non-malarial acute febrile illness (Non-Malarial AFI) and undiagnosed acute febrile illness (Undiagnosed AFI) (Aufi et al. 2021). Diagnosed AFI subtype refers to cases of AUFI where the underlying cause of fever has been identified and diagnosed whereby healthcare professionals would have successfully determined the specific pathogen or non-infectious condition (autoimmune disease, malignancy responsible for the fever. This will lead to application of appropriate treatment and management strategies to the patients. Meanwhile, non-malarial AFI subtype of AUFI excludes cases of fever caused by malaria. It encompasses a wide range of infectious and non-infectious causes of fever, including viral, bacterial, parasitic, autoimmune, and inflammatory conditions. Non-Malarial AFI often requires thorough diagnostic evaluation to identify the specific cause, and it can be challenging to differentiate between these various conditions based on clinical presentation alone. Undiagnosed AFI refers to cases where the cause of fever remains unclear or unknown even after a comprehensive diagnostic evaluation (Wangdi et al. 2019). Despite medical assessments and testing, the specific underlying condition responsible for the fever cannot be identified. These cases pose a diagnostic challenge and may require ongoing monitoring and further investigations as new information becomes available (Brown et al. 1984). The severity of acute AFI in the tropics and sub-tropics countries are now being recognized widely since there is a delay among medical community worldwide to reach the exact underlying causes of AUFI (Lokida et al. 2020). Hence it is essential to diagnosis febrile illness at early or at least intermediate stage since there are various reported infectious and noninfectious bacteria, viruses, fungus, and parasites that potentially trigger high fever among the patients. In addition, emerging new pathogen at the community level could also lead to poor point-of-care diagnosis of febrile illness. Table 1 displays the various subtype of AUFI.

**Table 1.** Various subtype of acute febrile illness.

Subtype	Description	Causes	Diagnosis	Management
		Specific pathogen or non-infectious condition (e.g. autoimmune disease, malignancy).	Healthcare professionals determine the cause and administer appropriate treatment.	
Diagnosed AFI	Underlying cause of fever identified.	Broad range of infectious and non-infectious causes (e.g. viral, bacterial, parasitic, autoimmune, inflammatory).	Requires thorough diagnostic evaluation due to difficulty in differentiating causes based on clinical presentation alone.	Treatment specific to the underlying cause.
Non-Malarial AFI	Fever not caused by malaria.	Treatment specific to the identified cause.		
Undiagnosed AFI	Cause of fever remains unclear after comprehensive evaluation.	Unknown underlying condition.	Ongoing monitoring and potential future investigations.	Symptomatic management and supportive care.

### AUFI and prevalence

Globally, the prevalence of AUFI is often higher in tropical and subtropical regions, this possibly due to a convergence of factors that create fertile ground for a diverse range of infectious diseases. These regions typically experience warm and humid climates, which provide an ideal environment for disease vectors like mosquitoes and ticks to thrive (Alexander et al. 2013). As a result, vector-borne diseases such as malaria, dengue fever, Zika virus, and various forms of encephalitis are endemic in these areas. The proliferation of vectors and their interactions with humans and animals contribute significantly to the transmission of febrile illnesses. Furthermore, tropical and subtropical regions often exhibit rich biodiversity and a close interface between humans, wildlife, and domestic animals. This ecological diversity increases the risk of zoonotic diseases, where infectious agents can jump from animals to humans. Wildlife species often serve as natural reservoirs for various pathogens, facilitating spillover events (Keesing and Ostfeld 2021). Limited access to healthcare, inadequate infrastructure, and socio-economic challenges in some of these regions can lead to delays in diagnosis and treatment, allowing infectious diseases to progress and become more severe. Climate change, which alters temperature and precipitation patterns, can shift the geographic distribution of disease vectors and pathogens, potentially expanding the prevalence of febrile illnesses. Hence AUFIs such as bacterial zoonotic diseases (scrub typhus and leptospirosis), respiratory tract infections, arboviral disease, mononucleosis, malaria, and typhoid fever have been commonly reported whereby more than 1 million leptospirosis, 5.2 million dengue, 5 million typhoid fever, 10 million malaria, 150.7 million of respiratory tract and 4 million mononucleosis cases have been diagnosed each year (Alexander et al. 2013, Keesing and Ostfeld 2021, Leal Filho et al. 2022). Another systematic reviews has demonstrated that the top infectious causes of AUFI were dengue fever (11.8% of cases), leptospirosis (4.4% of cases), typhoid (4.0% of cases), scrub typhus (4.0% of cases) and influenza other than H1N1 (3.1% of cases) in most countries in the South and Southeast Asia regions (Leal Filho et al. 2022). Among admitted patients, dengue fever was the primary cause of AUFI, while leptospirosis was the main cause for outpatients. The study also highlighted regional variations in the causes of AUFI. In South Asia, dengue fever was the main cause, while in Southeast Asia, leptospirosis was the primary diagnosis. Another paper also indicated that Burkina Faso and Sudan, both countries located in Sub-Saharan Africa, are mentioned as places where malaria is a particularly prevalent cause of AUFI in children. These regions have a high incidence of malaria cases due to the presence of malaria-transmitting mosquitoes. In addition to malaria, acute respiratory infections (ARIs) are noted as a primary cause of AUFI in Sub-Saharan Africa. ARIs encompass a range of respiratory illnesses, including pneumonia and bronchitis. These infections can lead to fever, cough, and difficulty breathing, making them a common cause of febrile illness (Ouédraogo et al. 2020). Another prospective observational study conducted among seven hospitals in India has demonstrated a mortality rate of 2.4% (37 deaths and 46.9% died within 2 days of admission, suggesting delayed hospitalization due to lack of clinical presentation as a contributing factor. Malaria, leptospirosis, scrub typhus, dengue fever and bacterial blood stream infections (including enteric fever) were to be most common causes in those fatal cases (Mørch et al. 2023). A case-control analysis from Vietnam has mentioned that out of 378 AUFI patients nearly 42.9% were undiagnosed AFI cases and bacterial infections and parasitic diseases bacterial infections and parasitic diseases, might be underrepresented (Le-Viet et al. 2019). Interestingly, an investigation has analysed that among 765 travelers returning from tropical and subtropical regions to Europe, 310 (40.5%) had a clear source of infection (mainly traveler's diarrhea or respiratory infections), while 455 (59.5%) were categorized as having AUFI. Over 40% of returning travelers with AUFI were diagnosed with malaria or dengue, infections that can be easily diagnosed by rapid diagnostic tests. This calls for development of new diagnostic tests and treatments for AUFI (Mørch et al. 2017). A study has reported that 340 patients with acute undifferentiated fever (AUF), 193 (56.8%) remained undiagnosed after extensive investigations, representing a significantly high prevalence of undiagnosed undifferentiated fever (UUDF) in Far North Queensland, Australia. After the prolonged diagnosis, dengue fever was the most frequent diagnosis (78 cases), followed by other infectious diseases like influenza, leptospirosis, and melioidosis. The study also revealed a higher incidence of UUDF during the rainy season (December to March), suggesting a potential link with increased mosquito activity and transmission of vector-borne diseases (Susilawati and McBride 2014).

However, clinicians believe that most of these cases are underdiagnosed and underreported as febrile illness hence the actual number is most likely much higher. A study has reviewed that the most common causative agent for AUFI cases in Peninsular Malaysia are dengue virus and *Leptospira* meanwhile malaria is more prevalent in Borneo. However, a recent study conducted in Teluk Intan, Perak at Peninsular Malaysia demonstrates that rickettsial infections are a significant cause of AFI whereby out of 309 hospitalized adults with AFI, 42 (19%) individuals were diagnosed with rickettsial infections and 4 (11%) patients with rickettsial infections died, highlighting the potential severity of this condition (Yuhana et al. 2022).

In addition, a 10 month period (2012 to 2013) cross-sectional study was conducted in University of Malaya Medical Centre (UMMC) with a collaboration of United States Naval Medical Research Unit 2 (NAMRU2), involving 119 adult inpatients from the infectious diseases ward. It was found that 67 (52.3%) cases of probable dengue and 25 (19.5%) cases of confirmed dengue, followed by typhus 8 (6.2%) cases, influenza 8 (6.2%) cases and leptospirosis 7(5.5%) cases have been reported using only serology testing which limits the diversity and relative importance of common infectious causes of AUFI (unpublished data). Table 2 shows the prevalence of AUFI worldwide.

**Table 2.** Acute febrile illness and prevalence.

Region	Most Common Causes of AUFI	Notes
South and Southeast Asia	Dengue Fever	11.8% of cases
	Leptospirosis	4.4% of cases
	Typhoid	4.0% of cases
	Scrub Typhus	4.0% of cases
	Influenza	3.1% of cases
	Respiratory Tract Infections	150.7 million cases
	Mononucleosis	4 million cases
	Malaria	10 million cases
	Bacterial Zoonotic Diseases	>1 million cases
Sub-Saharan Africa	Malaria	High incidence
	Acute Respiratory Infections (ARIs)	Common cause
Burkina Faso and Sudan	Malaria	Particularly prevalent cause in children
India	Malaria	Most common cause
	Leptospirosis	Most common cause
	Scrub Typhus	Common cause
	Dengue Fever	Common cause
	Bacterial Blood Stream Infections	Common cause
Vietnam	Bacterial Infections	Underrepresented
	Parasitic Diseases	Underrepresented
Europe	Malaria	40% of returning travelers with AUFI
	Dengue	40% of returning travelers with AUFI
Far North Queensland, Australia	Dengue Fever	Most frequent diagnosis after UUDF
	Influenza	Common cause
	Leptospirosis	Common cause
	Melioidosis	Common cause
	UUDF	56.8% of patients with AUF
Peninsular Malaysia	Dengue Virus	Most common cause
	Leptospira	Most common cause
	Rickettsial Infections	19% of hospitalized adults with AFI
	Dengue	52.3% of cases
	Typhus	6.2% of cases
	Influenza	6.2% of cases
	Leptospirosis	5.5% of cases

### AUFI and treatment

The treatment of AUFI depends on several factors, including the underlying cause of the fever, the severity of symptoms, and the patient's overall health. Since AUFI represents a broad category of illnesses, the treatment approach may vary widely. Firstly, the first step in treating AUFI is to identify the specific cause of the fever. This often requires a thorough medical history, physical examination, and diagnostic tests, which may include blood tests, imaging studies, and sometimes more specialized tests like serology or molecular tests for specific pathogens (Vanderschueren et al. 2003). While awaiting test results or if the cause remains unknown, supportive care is essential. This includes measures to relieve symptoms, maintain hydration, and reduce fever. Common interventions may include rest, adequate fluid intake, and over-the-counter medications like acetaminophen or ibuprofen to lower fever and alleviate discomfort (Bhaskaran et al. 2019). If a bacterial infection is suspected or confirmed, antibiotics may be prescribed to treat the specific bacteria responsible for the illness. The choice of antibiotics depends on the type of bacteria and its susceptibility to specific antibiotics. If a viral infection is diagnosed (e.g. influenza), antiviral medications may be used in some cases to reduce the severity and duration of symptoms (Gupta and Nischal 2020). However, not all viral infections have specific antiviral treatments. In regions where malaria is endemic, and if malaria is suspected or diagnosed, antimalarial medications are prescribed to treat the infection. In the case of dengue fever, which is a mosquito-borne viral illness, it's important to manage the patient's condition carefully. Hospitalization and supportive care are often necessary, as severe cases of dengue can be life-threatening (Rao et al. 2019). Patients with AUFI should be closely monitored for any worsening of symptoms, especially if they develop warning signs such as severe headache, difficulty breathing, chest pain, confusion, or persistent vomiting (Holgersson et al. 2022). Timely medical intervention may be needed.

### AUFI and preventive measure

Preventive measures for AUFI are essential in reducing the risk of contracting illnesses that result in fever and non-specific symptoms. Since AUFI encompasses a broad spectrum of diseases, adopting general preventative strategies is key. Begin with maintaining good hand hygiene by regularly washing hands with soap and water, especially after being in public spaces or before eating (Ovtcharenko and Oczkowski 2022). In cases where soap and water are not available, use hand sanitizer containing at least 60% alcohol. Staying up to date with recommended vaccinations is crucial, as it provides effective protection against numerous infectious diseases, including influenza, measles, mumps, rubella, hepatitis, and COVID-19 (Kullberg and Wiersinga 2023).

In regions where mosquito-borne illnesses like dengue fever and malaria are prevalent, preventing mosquito bites is paramount. Furthermore, addressing food and water safety is vital, involving safe food handling practices, thorough cooking, and the consumption of purified water in areas where waterborne diseases are a concern (Wiemer et al. 2017). In situations where contagion risk is high, such as during outbreaks or in healthcare settings, using appropriate personal protective equipment (PPE) like masks, gloves, and gowns is essential. It's equally important to avoid close contact with sick individuals, especially those displaying fever or symptoms of contagious illnesses. Practicing respiratory hygiene by covering the mouth and nose when coughing or sneezing helps prevent disease transmission. Maintaining a clean environment by regularly disinfecting frequently touched surfaces contributes to reducing the risk of infection. Table 3 shows potential intervention to control AUFI

**Table 3.** Potential intervention to control AUFI.

Intervention	Description	Benefits
Surveillance Programs	Monitoring human and animal populations for zoonotic pathogens that cause AUFI.	Early detection of outbreaks, identification of disease reservoirs and risk factors, evaluation of intervention effectiveness.
Veterinary Public Health	Implementing programs to control zoonotic diseases in animal populations.	Reduced risk of zoonotic transmission to humans, improved animal health and welfare.
One Health Education and Training	Training healthcare professionals, veterinarians, and environmental scientists in One Health principles and practices.	Improved awareness and understanding of zoonotic diseases, enhanced capacity for One Health collaboration.
Public Health Communication	Raising awareness among the public about zoonotic diseases and preventive measures.	Increased public knowledge, adoption of safe practices, reduced risk of exposure to zoonotic pathogens.
One Health Research	Conducting research on zoonotic diseases, including development of new diagnostics, vaccines, and treatment interventions.	Improved understanding of disease transmission and pathogenesis, development of effective tools for prevention, control, and treatment.
One Health Policy and Legislation	Developing policies and legislation to promote One Health approaches and address zoonotic diseases.	Improved coordination and collaboration across sectors, allocation of resources, effective legal framework for disease control.
Environmental Risk Management	Implementing measures to address environmental factors that contribute to zoonotic disease transmission.	Reduced risk of emergence and spread of zoonotic diseases, protection of ecosystems and biodiversity.
International Cooperation	Fostering collaboration among countries and international organizations to address zoonotic diseases.	Improved global disease surveillance and response, facilitated sharing of information and resources, equitable access to healthcare and interventions.

### The relationship between One Health and AUFI

The One Health approach is highly relevant when dealing with acute febrile illnesses, as it recognizes the interconnectedness of human health, animal health, and environmental health. Many AUFI are indeed zoonotic, which means they can be transmitted between animals and humans. These zoonotic diseases are caused by infectious agents, such as bacteria, viruses, parasites, and fungi that can jump from animals to humans and vice versa (Mackenzie and Jeggo 2019). These are examples to illustrate the diversity of zoonotic diseases that can lead to AUFI. First, influenza, commonly known as the flu, which known to infect various animal species, including birds and pigs. It frequently presents as an acute febrile illness. Influenza viruses are highly contagious and can lead to sudden fever, chills, body aches, and respiratory symptoms. Influenza shares many clinical symptoms with other acute febrile illnesses. This includes fever, cough, and muscle pain, which are common features of both influenza and other febrile illnesses. Influenza is known for causing seasonal outbreaks, commonly referred to as the flu season. During these periods, healthcare professionals should particularly vigilant for cases of acute febrile illnesses that might be attributed to influenza (Nayak 2014). Secondly, zoonotic coronaviruses such as COVID-19, caused by the novel coronavirus SARS-CoV-2. It is believed to have originated in bats and possibly passed to humans through an intermediate host. Both acute febrile illnesses and COVID-19 often present with fever as a prominent symptom. Fever is the body's response to infections and can be caused by a wide range of pathogens, including viruses, bacteria, and parasites. COVID-19 is primarily a respiratory illness and typically presents with symptoms such as cough, shortness of breath, and chest discomfort. While acute febrile illnesses can have respiratory symptoms, they may not always be as prominent as in COVID-19. The prevalence of COVID-19 in a community and a patient's exposure history are important considerations in diagnosing and managing febrile illnesses. Healthcare providers need to consider the possibility of COVID-19, especially during the ongoing pandemic (Alanagreh et al. 2020). Another one is rabies is a viral infection caused by the rabies virus, which is primarily transmitted through the saliva of infected animals, usually through bites or scratches. After being exposed to the rabies virus, a person may experience symptoms that are not specific to rabies, including fever, malaise, headache, and discomfort. These symptoms can resemble those of an acute febrile illness (Shepherd et al. 2023). Hantavirus Pulmonary Syndrome (HPS) is a severe respiratory illness caused by the Hantavirus. HPS typically begins with non-specific symptoms, including fever, muscle aches, fatigue, and sometimes gastrointestinal symptoms like nausea, vomiting, and abdominal pain. These symptoms can mimic those of an acute febrile illness caused by various pathogens. However, HPS is a potentially life-threatening disease, and its severity sets it apart from most cases of acute febrile illness. The rapid progression to respiratory failure and the risk of severe complications makes HPS a critical medical condition (Ramos 2008). On the other hand, brucellosis and acute febrile illness are related in the sense that brucellosis is a type of acute febrile illness characterized by fever, among other symptoms. Brucellosis, also known as undulant fever or Malta fever, is a bacterial infection caused by various species of the genus Brucella. Brucellosis is always considered one of the classic causes of acute febrile illness, especially in regions where the disease is endemic. Acute febrile illnesses are characterized by a sudden onset of fever and often involve non-specific symptoms such as fatigue, malaise, headache, and muscle aches, all of which can be present in brucellosis (Saddique et al. 2019). Leptospirosis is caused by the Leptospira bacteria and can vary in severity from mild to severe. Leptospirosis is classified as an acute febrile illness, which means it presents as a sudden and short-term feverish condition. Patients with leptospirosis often experience a rapid onset of symptoms, including fever, headache, and muscle pain (Haake and Levett 2015). Diagnosing this zoonotic disease can be challenging because its symptoms overlap with those of other febrile illnesses, such as dengue fever or influenza. Laboratory tests, including blood cultures and serological tests, are often needed for confirmation.

Febrile illnesses may be treated with antimicrobial agents, and the misuse of antibiotics refers to situations where antibiotics are used inappropriately or unnecessarily. This can include taking antibiotics for viral infections (common cold or flu), using antibiotics without a prescription, not completing the full course of prescribed antibiotics, or using antibiotics in animal agriculture for growth promotion rather than disease treatment (Haake and Levett 2015). By promoting responsible antimicrobial use in both healthcare sectors and by addressing AMR through a One Health approach, it is possible to mitigate the development and spread of antimicrobial resistance, ensuring that antibiotics remain effective for treating bacterial infections in the future. One Health can help improve disease surveillance involves the coordinated collection, analysis, and interpretation of health data from multiple sources, including human health, animal health, and environmental data, prevention, and control strategies for AUFI, ultimately benefiting both human and animal populations and the environment (Nadjm et al. 2012, Ajuwon et al. 2021, Blair et al. 2022, Grundy and Houpt 2022, Raab et al. 2022).

### Emerging Zoonotic Risks and Ongoing Outbreaks

The threat of novel zoonotic diseases and ongoing outbreaks COVID-19 and its potential evolution towards endemicity, due to overlapping symptoms and difficulty in rapid diagnosis, this adds a complex layer to the already challenging landscape of AUFI. The emerging zoonotic risks are due to increased human encroachment on wildlife habitats and deforestation heighten the risk of zoonotic pathogens jumping from animals to humans (Meurens et al. 2021). New viral strains, bacterial zoonoses, and even fungal infections could present as AUFI, posing diagnostic challenges (Bardhan et al. 2023). In addition, Climate change is one of the greatest threats to human health in the 21st century. Shifting temperature and precipitation patterns can alter the geographic distribution of vector-borne diseases like arboviruses, expanding their reach and contributing to AUFI cases in previously unaffected regions (Caminade et al. 2019). Lastly, the intensification of animal agriculture and close contact with domesticated animals create additional pathways for zoonotic transmission. Emerging pathogens originating in livestock may manifest as AUFI in human populations (Jones et al. 2013). On top of that, the emergence of antimicrobial resistance in pathogens causing common AUFI-associated diseases like leptospirosis and enteric fever can complicate treatment and lead to prolonged illness, contributing to undiagnosed or misdiagnosed cases. In addition, incomplete or insufficient vaccine coverage for established zoonotic diseases like influenza and rabies can leave populations vulnerable to outbreaks, leading to an upsurge in AUFI cases with similar presentations (Trott et al. 2018).

**Figure 1.**
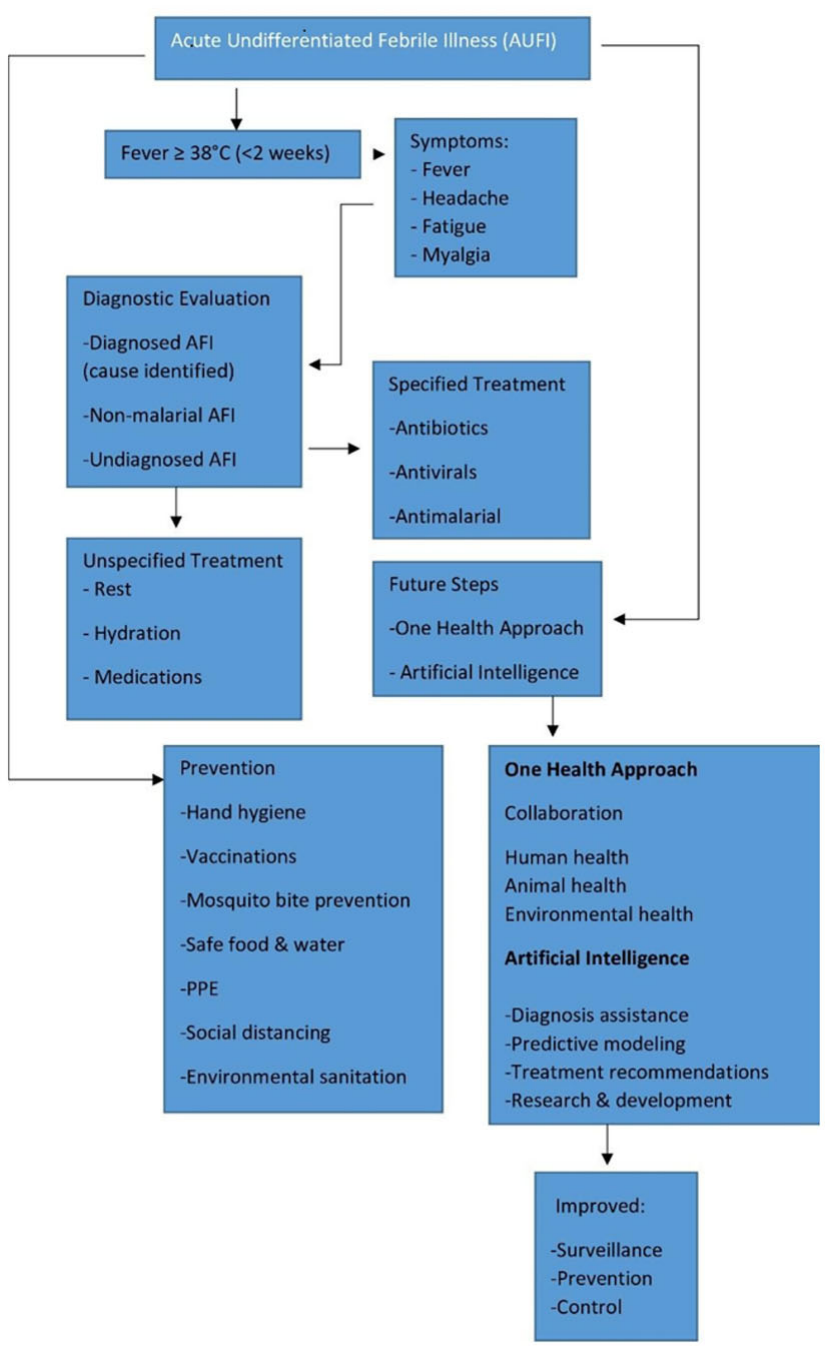
AUFI: A complex clinical presentation.

Emerging coronaviruses is one the example of zoonotic disease including SARS-CoV-2, can jump from animals to humans through various contacts and adapt to human transmission. SARS-CoV-2 is a novel coronavirus that was identified as the cause of the COVID-19 pandemic, which began in late 2019 (Haider et al. 2020). SARS-CoV-2 has demonstrated the ability to mutate and develop new variants, some of which may impact transmissibility, severity, or immune evasion. Continuous monitoring of variants is crucial for understanding their potential impact on public health. Another example, enterohemorrhagic *Escherichia coli* (E. coli) O157:H7 is a strain of E. coli bacteria that can cause severe illness, particularly through foodborne transmission. It stands out due to its association with severe complications such as hemolytic uremic syndrome, renal failure, and a significant contribution to foodborne outbreaks worldwide. Outbreaks associated with this strain have been reported worldwide, often linked to consumption of contaminated food, including leafy greens like spinach or lettuce. The bacteria can contaminate these foods during production, processing, or handling (Lim et al. 2010). Rickettsia is a genus of bacteria that includes several species responsible for causing diseases in humans, such as Rocky Mountain spotted fever, typhus, and rickettsial pox. Changes in climate patterns, including temperature, humidity, and precipitation, can influence the distribution and behavior of vectors like ticks, fleas, and mites that transmit Rickettsia bacteria. These alterations in environmental conditions might expand the geographical range of these vectors, potentially increasing the incidence of Rickettsia-related diseases in new areas or elevations where they were previously uncommon (Blanton 2019). Meanwhile, Henipavirus: belongs to the Henipavirus genus, also responsible for Nipah and Hendra viruses known for their high mortality rates. Recent cases in China suggest animal-to-human transmission, with shrews identified as potential reservoirs. The virus is newly discovered, and much remains unknown about its transmission dynamics, pathogenicity, and long-term consequences (Quarleri et al. 2022). The outbreak of monkeypox outside its usual endemic regions in Africa during 2022–2023 indeed raised significant concerns in the public health sphere. Monkeypox is a rare viral zoonotic disease that is primarily found in Central and West African countries. The outbreak outside of these regions alerted health authorities globally about the potential for the disease to spread beyond its endemic area. The outbreak highlighted the possibility of zoonotic spillover events, where the virus can jump from animals to humans, potentially through contact with infected animals or contaminated animal products (Antunes and Virgolino 2022). During the COVID-19 pandemic, mucormycosis, a rare but severe fungal infection primarily affecting immunocompromised individuals, witnessed a concerning surge, notably among COVID-19 patients. The infection's escalation was attributed to compromised immune systems due to COVID-19 severity, corticosteroid treatments, and various medical interventions, emphasizing the susceptibility to opportunistic infections. Heightened awareness among healthcare providers became crucial for early identification of mucormycosis symptoms—facial swelling, nasal congestion, and black lesions—and prompt initiation of appropriate antifungal therapies and, if necessary, surgical interventions (Dam et al. 2023). Meanwhile, melioidosis is a soil- and water-borne bacterium is causing increasing infections in Southeast Asia and Northern Australia, causing pneumonia, abscesses, and even death. Brucellosis bacterial disease transmitted through contact with livestock (mainly cattle) can cause chronic fatigue, fever, and joint pain. Stronger public health measures and livestock vaccination programs are necessary for control (Boone et al. 2017). Figure 1 displays AUFI complex clinical presentation.

### Challenges in resource-limited settings handling AUFI

**Figure 2.**
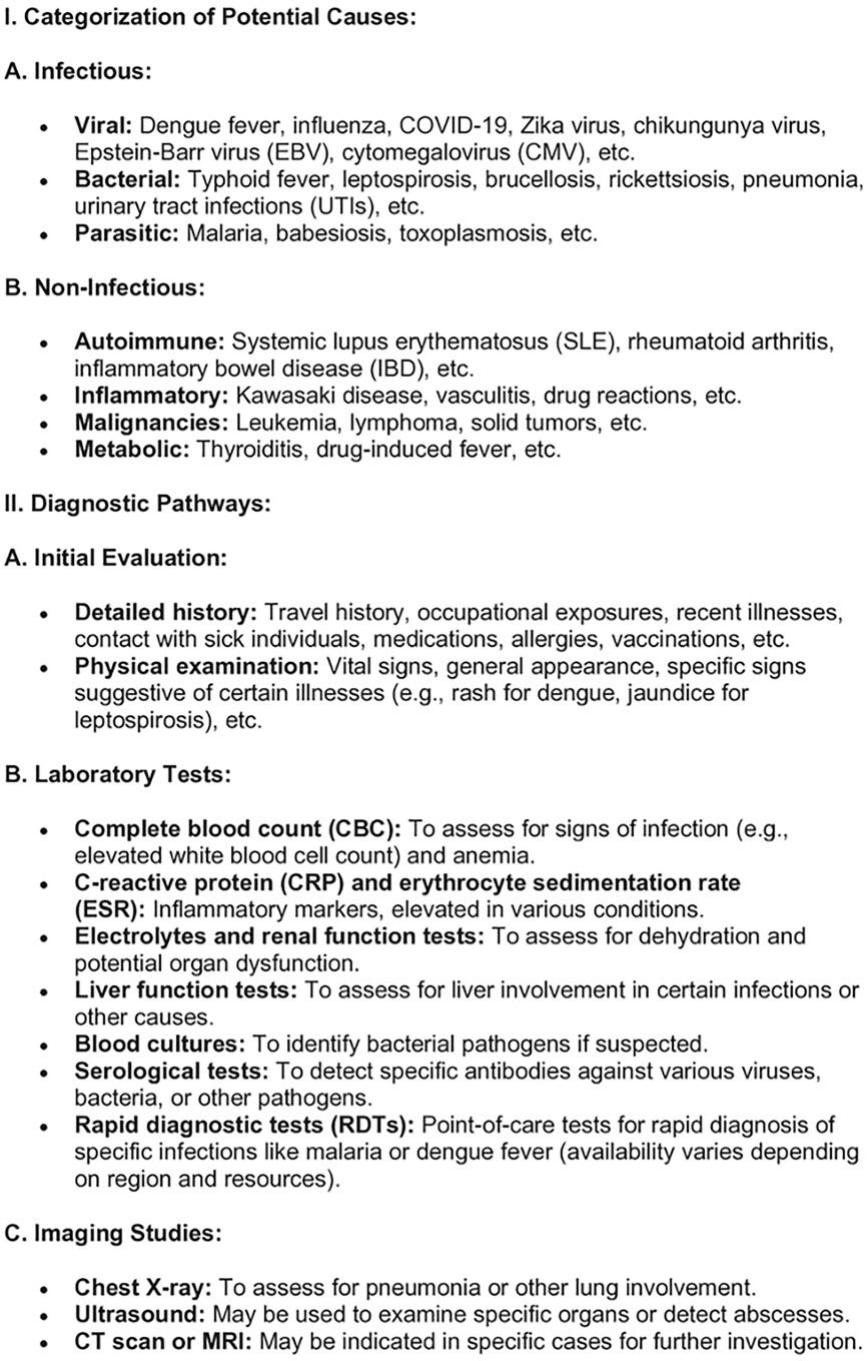

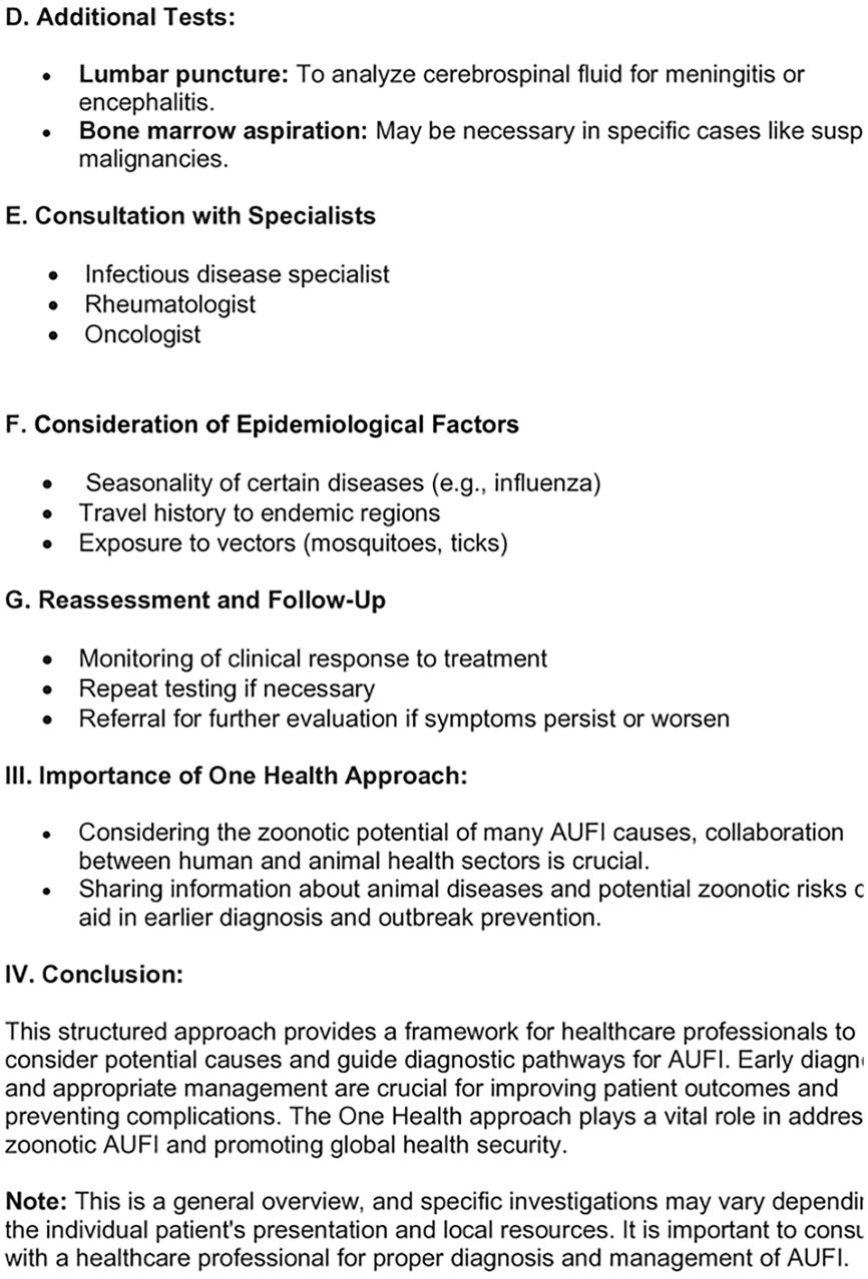
This flowchart provides a structured approach to diagnosing AUFI by considering various potential causes and guiding clinicians through the diagnostic process based on clinical presentation, epidemiological factors, and laboratory findings.

Resource-limited settings grapple with multifaceted challenges in dealing with emerging zoonotic diseases, significantly impeding effective healthcare access, diagnosis, treatment, public health interventions, and overall disease management. The dearth of healthcare infrastructure, scarcity of medical facilities, and a critical shortage of healthcare personnel hinder timely diagnosis and provision of care, exacerbating the challenges faced by patients seeking essential healthcare services (Shiferaw et al. 2017). Diagnostic difficulties arise from the absence of advanced laboratory facilities and rapid tests, compounded by limited awareness among healthcare workers about emerging zoonotic diseases and overlapping symptoms with more common ailments, leading to misdiagnoses and delayed treatments. Compounding this issue, limited access to specific antiviral, antibacterial, or antiparasitic medications and inadequate supportive care due to financial constraints significantly hamper effective treatment, leaving many patients untreated or facing suboptimal therapeutic options (John et al. 2008). Weak disease surveillance systems, coupled with insufficient public awareness and education about zoonotic diseases, impede early detection, outbreak control, and preventive measures, allowing these diseases to spread undetected (Villarroel et al. 2023). Moreover, fragmented communication and collaboration between human and animal health sectors hinder the implementation of effective One Health strategies, limiting preventive measures and exacerbating disease transmission. These challenges result in undiagnosed cases, delayed treatments, increased disease transmission, and overwhelming strain on already overburdened healthcare systems, ultimately impacting patient outcomes and escalating mortality rates (Ghai et al. 2022). Addressing these complex challenges demands a concerted effort toward bolstering healthcare infrastructure, enhancing diagnostic capabilities, expanding healthcare workforce, ensuring access to affordable medications and supportive care, strengthening disease surveillance, conducting robust public awareness campaigns, and fostering effective collaboration between human and animal health sectors through a comprehensive One Health approach, crucial for mitigating the devastating impact of emerging zoonotic diseases in resource-limited settings and curbing their widespread transmission. Figure 2 provides the overall flowchart on a structured approach to diagnosing AUFI by considering various potential causes and guiding clinicians through the diagnostic process based on clinical presentation, epidemiological factors, and laboratory findings.

## Conclusion

The challenge of Acute Undifferentiated Febrile Illness (AUFI) encompasses a complex landscape of diverse causes, diagnostic hurdles, and treatment intricacies, particularly accentuated in resource-limited settings. This condition, characterized by fever and nonspecific symptoms, presents a diagnostic puzzle often compounded by limited healthcare access, inadequate diagnostic tools, and overlapping disease manifestations. The multifaceted subtypes within AUFI demand tailored approaches for Diagnosed AFI, Non-Malarial AFI, and Undiagnosed AFI, emphasizing the critical need for improved diagnostics and ongoing research. Mitigating AUFI's impact requires a multifaceted strategy incorporating preventive measures, enhanced healthcare infrastructure, heightened disease surveillance, interdisciplinary collaboration under the One Health framework, and a concerted effort to address emerging zoonotic risks and ongoing outbreaks. A comprehensive approach is vital for effectively managing AUFI, ensuring timely diagnosis, appropriate treatment, and preventive measures, especially crucial in resource-limited regions where healthcare challenges are most pronounced.

## Ethics declarations

Not applicable.

## Consent for publication

Not applicable.

## Availability of data and materials

Not applicable.
